# *Akkermansia muciniphila* is Negatively Correlated with Hemoglobin A1c in Refractory Diabetes

**DOI:** 10.3390/microorganisms8091360

**Published:** 2020-09-05

**Authors:** Ching-Tang Shih, Yao-Tsung Yeh, Ching-Chiang Lin, Lin-Yu Yang, Chih-Po Chiang

**Affiliations:** 1 Department of Family Medicine, Fooyin University Hospital, Pingtung 92849, Taiwan; aaronshih@hotmail.com; 2 Department of Education and Research, Fooyin University Hospital, Pingtung 92849, Taiwan; glycosamine@yahoo.com.tw (Y.-T.Y.); x6053@ms25.hinet.net (C.-C.L.); 3 Department of Medical Laboratory Sciences and Biotechnology, Fooyin University, Kaohsiung 83102, Taiwan; 4 Aging and Disease Prevention Research Center, Fooyin University, Kaohsiung 83102, Taiwan; 5 Department of Community Health Center, Fooyin University Hospital, Pingtung 92849, Taiwan; 1200570@fy.org.tw; 6 Department of Surgery, Kaohsiung Medical University Hospital, Kaohsiung 80756, Taiwan; 7 Division of Breast Surgery, Department of Surgery, Kaohsiung Medical University Hospital, Kaohsiung 80756, Taiwan

**Keywords:** gut microbiota, type 2 diabetes, refractory diabetes, hemoglobin A1C, *Akkermansia muciniphila*

## Abstract

Patients with refractory diabetes are defined as type 2 diabetes (T2D) patients; they cannot achieve optimal glycemic control and exhibit persistent elevations of hemoglobin A1c (HbA1c) ≥8% while on appropriate therapy. Hyperglycemia can lead to severe microvascular/macrovascular complications. However, in contrast to T2D, few studies have focused specifically on the gut microbiota in refractory diabetes. To examine this issue, we recruited 79 subjects with T2D and refractory diabetes (RT2D), and all subjects received standard therapy with Metformin or other hypoglycemic agents with or without insulin for at least one year. The α-diversity displayed no significant difference, whereas the β-diversity showed a marginal significance (*p* = 0.054) between T2D and RT2D. The evaluation of taxonomic indices revealed reductions in both *Akkermansia muciniphila* and *Fusobacterium* and a corresponding enrichment of *Bacteroides vulgatus, Veillonella denticariosi* among those with RT2D. These microbial markers distinguished RT2D from T2D with an acceptable degree of discrimination (area under the curve (AUC) = 0.719, *p* < 0.01) and were involved in several glucose-related functional pathways. Furthermore, the relative abundance of *Akkermansia muciniphila* was negatively correlated with HbA1c. Our combined results reveal unique features of the gut microbiota in RT2D and suggest that the evaluation of the gut microbiota could provide insights into the mechanisms underlying glycemic control and the impact of therapeutic modalities in patients with RT2D.

## 1. Introduction

According to recent reports from the World Health Organization, 422 million people worldwide carried a diagnosis of type 2 diabetes (T2D) in 2014, and this number is expected to reach ~552 million in 2030, which will mean that this condition will rank as the seventh leading cause of death worldwide [1,2]. T2D is a metabolic disease associated with dysregulated glucose metabolism; patients typically present with insulin resistance or reduced insulin secretion, meaning that blood glucose levels are not maintained at appropriate levels. Patients with T2D typically experience both microvascular and macrovascular complications. Microvascular complications include retinopathy, nephropathy and neuropathy, whereas macrovascular complications can lead to cardiovascular disease [3]. Approximately 40% of T2D patients will develop microvascular complications, which is the major cause of renal failure and blindness associated with this condition [4,5,6]. Hyperglycemia is a major risk factor for developing both microvascular and macrovascular complications associated with T2D; as such, good glycemic control remains a critical issue. Patients with refractory T2D (RT2D) are those who cannot maintain good glycemic control, with persistent elevations in hemoglobin A1c (HbA1c) ≥8% even with appropriate therapy [7,8]. Only a few studies have described refractory diabetes, and the major causes of refractory diabetes come from the environment of specialist centers, poor healthcare, a lack of adherence to treatment and clinical inertia [7,9,10].

Diabetes is a complex metabolic disorder that is closely related to diet, an obese lifestyle and lack of exercise [11]. Numerous studies have implicated alterations in the gut microbiota as contributing to the development of T2D; specifically, dysbiosis was reported to promote increased insulin resistance, together with an associated impact on metabolism and glucose levels [12,13,14]. Lipopolysaccharide (LPS), also known as endotoxin, is a prominent constituent of Gram-negative bacteria and interacts with the complex of CD14 and the Toll-like receptor 4 (TLR4) at the surface of innate immune cells. LPS induces the secretion of proinflammatory cytokines and inflammation, resulting in lipogenesis and the reduced sensitivity of insulin [15]. Secondary bile acids produced by specific gut bacteria also have an impact on host glucose levels [16,17]. The gut microbiota will also digest dietary fiber into short-chain fatty acid (SCFA), in which butyric acid will promote beta-cells to secrete insulin and regulate the homeostasis of glucose levels [18]. Furthermore, the results of a recent study revealed that changes in the gut microbiota that can be detected before the onset of T2D may potentially be useful for early diagnosis and intervention [19].

Thus, in this study, we aimed to provide a characterization of the gut microbiota in RT2D patients. Only a few studies have focused specifically on the mechanisms associated with the development of RT2D; furthermore, even fewer studies have focused on the gut microbiota associated with RT2D. We hypothesize that the gut microbiota may be involved in refractory diabetes and contribute to glycemic control, providing a novel therapeutic approach for refractory diabetes patients.

## 2. Materials and Methods

### 2.1. Patient Recruitment

A total of 79 subjects were recruited from the Department of Family Medicine. These subjects suffered from type 2 diabetes (T2D) and had undergone standard oral glucose-lowering drugs (OGLDs) therapy with or without insulin for at least one year. Metformin was the first-line therapy for all T2D patients except where contraindicated by renal dysfunction. Additional OGLDs such as Sulfonylureas, Dipeptidyl peptidase-4 (DPP-4) inhibitors, Alpha-glucosidase inhibitors and Thiazolidinediones were added to Metformin if required to achieve glycemic control. Long-acting insulin Detemir was provided if Metformin and OGLDs combined could not achieve glycemic goals. The drug adjustment protocol followed the recommendations from the American Diabetes Association standards of medical care in diabetes. All subjects were divided into two groups according to hemoglobin A1C (HbA1c): T2D with good glycemic control of HbA1c <8% (N = 52, mean age 66.38 ± 1.314 years) and refractory diabetes (RT2D) with poor glycemic control of HbA1c ≥8% (N = 27, mean age 64.37 ± 2.194 years). Fecal samples were collected for DNA extraction, and biochemical indexes at collection points were also recorded. All subjects were approved by the Internal Review Board and informed consent was obtained from all subjects (FYH-IRB-106-05-02).

### 2.2. Fecal DNA Extraction

Genomic DNA was extracted from the fecal samples with the QIAamp Fast DNA Stool Mini Kit (Qiagen, Germantown, MD, USA) with a modification of the manufacturer’s instructions. Briefly, the stool was lysed with the inhibitEX buffer and homogenized with FastPrep-24 5G (MP biomedicals, Irvine, CA, USA). Proteinase K and ethanol were added, and the supernatant was then processed with the QIAamp spin column and eluted with a pre-heated elution buffer. DNA concentrations were evaluated using a NanoDrop 2000 spectrophotometer.

### 2.3. PCR Amplification and 16S Sequencing

The library was constructed via the amplification of the standard V3-V4 region of the 16S rRNA gene. PCR was amplified with KAPA HiFi hotstart readymix (Roche, Branchburg, NJ, USA) following the instructions of Illumina 16S metagenomics sequencing library preparation. The PCR product was further purified with AMPure XP magnetic beads (Beckman Coulter, Brea, IN, USA) and barcoded by the Nextera XT index kit (Illumina, San Diego, CA, USA). The quality of the amplification products was evaluated with a Fragment Analyzer (Advanced Analytical, Ankeny, IA, USA) and quantified by the Qubit dsDNA HS assay kit (Life Technologies, Pleasanton, CA, USA). The PhiX control (20%) was added to the final pool of 10 pM, and the library was sequenced on a MiSeq (Illumina, San Diego, CA, USA) with the paired-end reads (2 × 300 nt) using a MiSeq Reagent Kit V3 for 600 cycles. Approximately 800 (K/mm^2^) clusters were generated, with over 90% passing the filter with Q30 ≥ 80% and at least 50,000 reads per sample. FASTQ files were collected and subjected to further analysis [20].

### 2.4. Bioinformatics Analysis

The raw paired-end reads were trimmed and passed through quality filters (quality trimming, discarding short read length and removing chimeras) and were assigned to operational taxonomic units (OTUs) which shared ≥97% similarity with the Greengene database. The raw paired-end reads were also analyzed with the basespace Ribosomal Database Project (RDP) classifier. Operational taxonomic units (relative abundance, heatmap, Krona and differential abundance analysis), α-diversity (Shannon index), and β-diversity (PCoA-Unweighted UniFrac) were determined with basespace (Illumina, San Diego, CA, USA), CLC Microbial Genomics Module (Qiagen, Germantown, MD, USA) and Graphpad Prism 7 (GraphPad Software, La Jolla, CA, USA). The OTU table was generated by CLC Microbial Genomics Module to be further analyzed with the linear discriminant analysis effect size (LEfSe) and for Phylogenetic Investigation of Communities by Reconstruction of Unobserved States (PICRUSt) analysis. LEfSe was conducted by Galaxy/HutLab to identify specific microbial markers between groups, with an alpha value for the factorial Kruskal–Wallis test/pairwise Wilcoxon test of 0.05 and an LDA score cut-off of 2.0. PICRUSt prediction was conducted by Galaxy/HutLab according to the Kyoto Encyclopedia of Genes and Genomes (KEGG) functional pathways database and analyzed with the Statistical Analysis of Metagenomic Profiles (STAMP) software. The STAMP criteria were set up, removing unclassified reads, with *p* < 0.01 and an effect size of 1. The results identified functional pathways with a significantly different abundance at level 3 between groups [20].

### 2.5. Statistical Analysis

A comparison of different groups was performed by the two-tailed *t*-test. Values of *p* less than *p* < 0.05, *p* *<* 0.01 and *p* < 0.001 were considered to be statistically significant. The specificity and sensitivity of the microbial markers were determined with the receiver operating characteristic curve (ROC curve) and the area under the curve (AUC) value. Correlations were calculated as the Pearson correlation coefficient [20].

## 3. Results

### 3.1. Characteristics of the Study Subjects and Diversity between T2D and RT2D

In this study, we recruited 79 participants who were divided into two groups according to HbA1c. These groups included those patients with T2D who maintained good glycemic control (HbA1c <8%) and those patients with RT2D who were unable to maintain glycemic control, as indicated by HbA1c ≥8%. All study participants received standard oral glucose-lowering drugs (OGLDs) therapy with or without insulin for at least one year, and we collected biochemical indexes and feces for analysis. The biochemical indexes and general characteristics of the individuals in these two groups are summarized in Table 1. As shown, the values for HbA1c and glucose Ante Cibum (glucose AC) were significantly different between T2D and RT2D. The mean HbA1c for T2D patients was 7.03% vs. 8.99% for RT2D patients (*p* < 0.001), and glucose AC for T2D patients was 135.1 mg/dL vs. 158.9 mg/dL for RT2D patients (*p* = 0.03). Nearly all study participants were treated with Metformin as first-line standard therapy except for patients who presented with decreased renal function.

**Figure 1:**
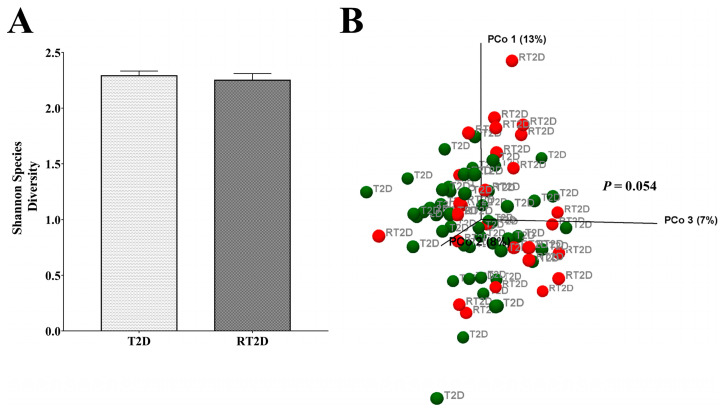
The microbial diversity of different groups. (**A**) No significant differences were observed concerning the α-diversity (Shannon index) when comparing microbial communities from T2D and RT2D. (**B**) The β-diversity of the principal coordinate analysis (PCoA) demonstrated marginally significant differences (*p* = 0.054) in the total microbial composition between T2D and RT2D groups.

Initially, we analyzed the α and β-diversity between T2D and RT2D patients. The α-diversity of the Shannon index indicated the diversity of the microbial community of different groups, and there were no significant differences between T2D and RT2D (Figure 1A). However, in terms of the β-diversity, the principal coordinate analysis (PCoA) of Unweighted UniFrac was assessed to evaluate the total microbial composition between different groups. The PERMANOVA test revealed marginally significant differences (*p* = 0.054) in the overall microbial composition between T2D and RT2D (Figure 1B); thus, we conclude that the gut microbial communities from participants diagnosed with T2D and RT2D displayed a similar α-diversity but slightly different β-diversity.

### 3.2. Relative Abundances of Verrucomicrobia and Fusobacteria Were Reduced in RT2D

**Figure 2:**
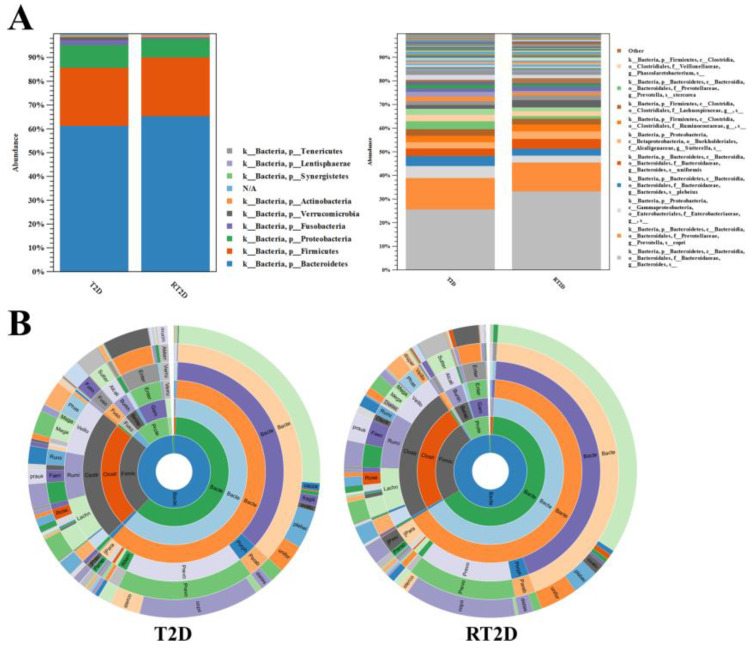
Operational taxonomic units (OTUs) at the phylum and species level between T2D and RT2D. (**A**) The relative abundance of OTUs at the phylum and species level in T2D and RT2D groups. (**B**) Krona charts revealed that the relative abundance of *Bacteroide* was increased, and *A*. *muciniphila* and *Fusobacterium* were diminished in the samples from participants diagnosed with RT2D.

The evaluation of the β-diversity of the total microbial composition revealed marginally significant differences between samples from the patients with T2D vs. those with RT2D. This was evaluated further with a focus on operational taxonomic units (OTUs). At the phylum level, the majority of OTUs were *Bacteroidetes* and were slightly increased in RT2D. Furthermore, the relative abundance of *Verrucomicrobia* and *Fusobacteria* was reduced in the RT2D group (Figure 2A). This tendency was observed more clearly in the Krona charts, where the species of *Verrucomicrobia*--*Akkermansia muciniphila* (*A*. *muciniphila)* was reduced in RT2D (Figure 2B).

### 3.3. Microbial Markers in T2D and RT2D Patients

**Figure 3:**
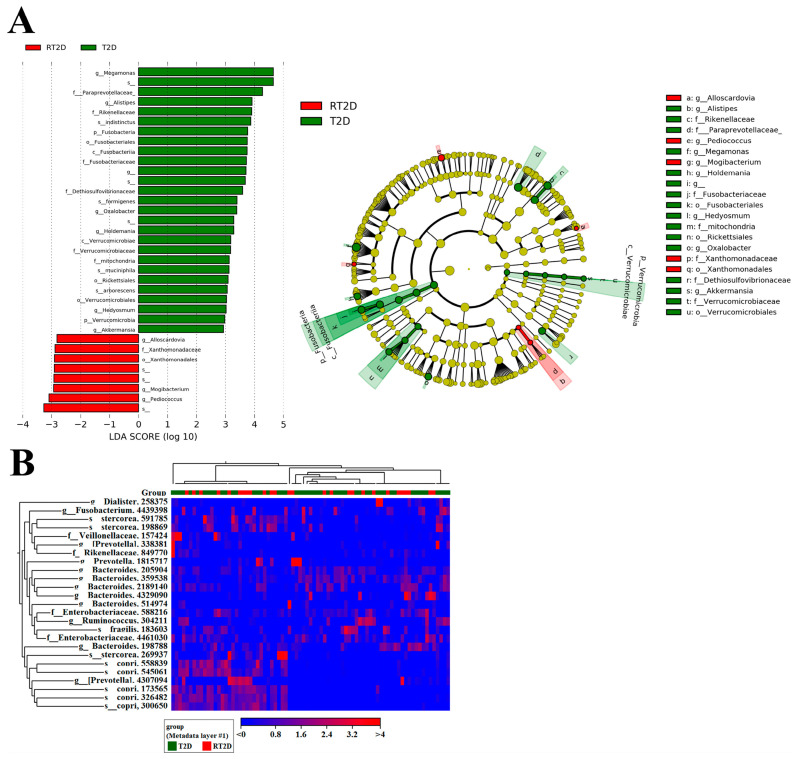
Microbial markers that distinguish between T2D and RT2D. (**A**) linear discriminant analysis effect size (LEfSe) analysis revealed that *Verrucomicrobia, A*. *muciniphila* and *Fusobacteria* were more abundant in T2D patients. (**B**) Heatmap analysis representing relatively high (red) and low (blue) abundances of specific microbial constituents in fecal samples from T2D and RT2D patients.

To further elucidate the microbial markers associated with T2D and RT2D patients, we analyzed the OTUs with the linear discriminant analysis effect size (LEfSe) and heatmap analysis. The input criteria of LEfSe analysis included an LDA score >2 and an alpha value for the factorial Kruskal–Wallis test/pairwise Wilcoxon test of 0.01. We focused specifically on the genus/species level and, in an identical manner to the previous abundance of OTUs, *Verrucomicrobia, A*. *muciniphila* and *Fusobacteria* were enriched in T2D patients (Figure 3A). We also conducted a heatmap analysis with the relative richness of the microbiome in high (red) and low (blue) between T2D and RT2D. Therefore, several potential microbial markers including *Verrucomicrobia, A*. *muciniphila* and *Fusobacteria* were revealed in T2D patients (Figure 3B).

### 3.4. Akkermansia Muciniphila Was Negatively Correlated with HbA1c

**Figure 4:**
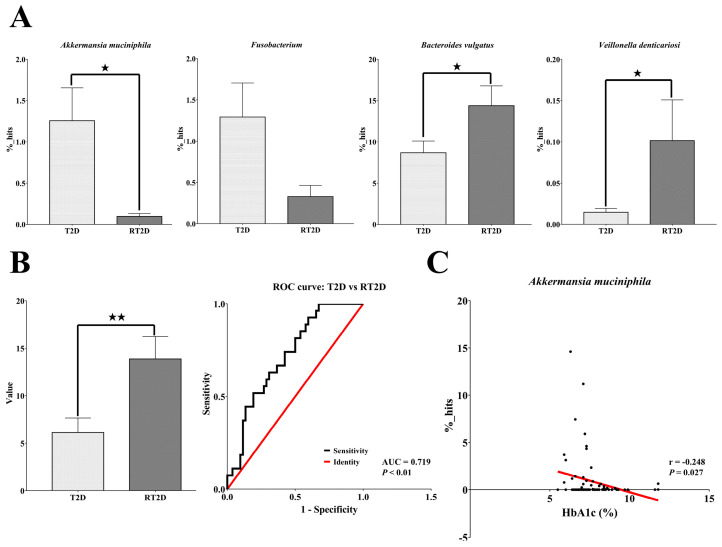
Specific glucose-related microbial markers identified in T2D and RT2D patients (**A**) Microbial markers from LEfSe and heatmap analysis were reconfirmed by Ribosomal Database Project (RDP) classification. As shown, the relative proportions of *A. muciniphila* and *Fusobacterium* were reduced and those of *B. vulgatus* and *V. denticariosi* were significantly increased in fecal samples from RT2D patients. (**B**) The average value from the percentages of *Bacteroides vulgatus* minus the sum of the percentages of *A*. *muciniphila, Fusobacterium* and *Veillonella denticariosi* were used to distinguish RT2D patients from T2D patients (area under the curve (AUC) = 0.719). (**C**) The relative proportion of *A*. *muciniphila* was significantly negatively correlated with HbA1c. A *p* value less than ★ *p* < 0.05 and ★★ *p* < 0.01 indicates statistical significance.

To further elucidate the microbial markers, we utilized the basespace of the Ribosomal Database Project (RDP) classifier to reconfirm the OTUs at the genus/species level. Using the RDP classifier, we screened all of the potential microbial markers revealed by LEfSe and heatmap analysis at the genus/species level; we eliminated the microbiomes with low percentages or that were not present in statistically significant numbers in both T2D and RT2D patients. Eventually, we identified four bacterial taxa that were abundant and/or present in statistically significant quantities in samples from both T2D and RT2D patients. Compared with T2D patients, the relative abundances of *A*. *muciniphila* (T2D: 1.25%; RT2D: 0.09%, *p* = 0.04) and *Fusobacterium* (T2D: 1.29%; RT2D: 0.33%, *p* = 0.10) were reduced in patients with RT2D; in contrast, the relative abundances of *Bacteroides vulgatus* (T2D: 8.68%; RT2D: 14.34%, *p* = 0.03) and *Veillonella denticariosi* (T2D: 0.01%; RT2D: 0.10%, *p* = 0.01) were significantly increased in the RT2D patient cohort (Figure 4A).

We further calculated a value representing the percentage of *Bacteroides vulgatus* minus the sum of the percentages of *A*. *muciniphila, Fusobacterium* and *Veillonella denticariosi.* This average value (T2D: 6.12 versus RT2D: 13.86, *p* < 0.01) was evaluated further using the receiver operating characteristic curve (ROC curve). The area under the curve (AUC) was 0.719 *(p* < 0.01), displaying acceptable discrimination for use in distinguishing patients with RT2D from those with T2D (Figure 4B). Equally importantly, we found that the relative proportion of *A*. *muciniphila* was significantly negatively correlated with levels of HbA1c (*r* = −0.248, *p* = 0.02, Figure 4C). These results reveal that *A*. *muciniphila* is a unique glucose-related microbial marker that reflects the clinical status of both T2D and RT2D patients.

### 3.5. Microbiome-Related Functional Pathways between T2D and RT2D Patients

**Figure 5:**
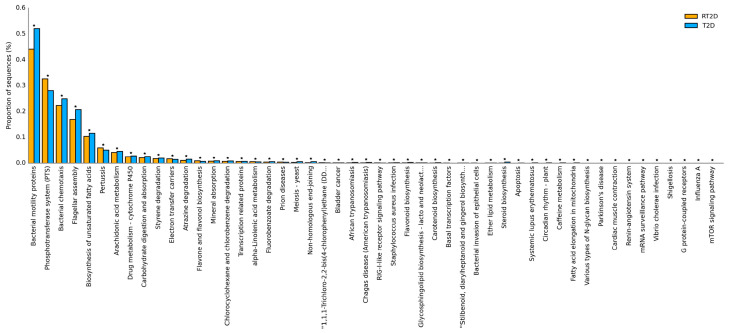
The potential functional pathway involved in T2D and RT2D patients. Functional pathways contributing to the biosynthesis of unsaturated fatty acids and arachidonic acid metabolism were reduced and those associated with the phosphotransferase system (PTS) were increased in RT2D patients. ^★^ indicates statistical significance.

To further determine the function of the microbiome in T2D and RT2D patients, we used a PICRUSt prediction of different proportions of the microbiome against the KEGG level 3 pathway; the criteria included the removal of unclassified reads, a value of *p* <0.05 and an effect size of 1 from STAMP software. We identified 50 functional pathways that were enriched in RT2D versus T2D, and most pathways were too small to be considered further; thus, we concentrated on those with a proportion of ≥0.05%. Compared with the results from T2D patients, pathways contributing to the proportion of the microbiome against the biosynthesis of unsaturated fatty acids and arachidonic acid metabolism were reduced, and those associated with the phosphotransferase system (PTS) were increased in RT2D patients (Figure 5). These results indicated that the differences observed concerning the microbiome content might have an impact on several critical functional pathways.

## 4. Discussion

Several studies that were published over the past few years have provided insight into the relationships between the gut microbiota and diabetes. Among these findings, specific groups and/or species of the gut microbiota were found to increase the endotoxemia of lipopolysaccharides (LPS), which can induce the secretion of proinflammatory cytokines and can promote insulin resistance and diabetes [21,22,23]; this information suggested a crucial link between dysbiosis and the development of T2D. Similarly, secondary bile acids [16,17] and short-chain fatty acid (SCFA) [18] also influence metabolism and homeostatic blood glucose levels [24]. The dynamic and adjustable nature of the gut microbiota may provide important information concerning the early detection and prevention of T2D as well as the current level of glycemic control.

However, only a few studies have described refractory diabetes, and studies on the relationship between gut microbiota and refractory diabetes are limited. The environment plays a large role in RT2D; this condition may occur more frequently within specialty practices than in primary care clinics, as a specialist physician may not have frequent contact with all patients [7,9]. Furthermore, poor healthcare, a lack of adherence to treatment and clinical inertia have all been suggested as reasons underlying the development of refractory disease. In 2014, one crucial study found a type of refractory hyperglycemia after Roux-en-Y gastric bypass (RYGB) surgery, indicating that the gut microbiota may be intimately involved in the development of RT2D [25,26]. In our studies, the microbial diversity in terms of the Shannon index showed no significant difference, but the total microbial composition revealed marginally significant differences (*p* = 0.054) between T2D and RT2D, indicating that the microbial diversity may not be a critical factor, but that specific species may be involved in the pathogenesis of RT2D disease.

To this end, our results revealed a reduced abundance of *Fusobacterium* (T2D: 1.29%; RT2D: 0.33%, *p* = 0.10) and an increased abundance of *Veillonella denticariosi* (T2D: 0.01%; RT2D: 0.10%, *p* = 0.01) in fecal samples from RT2D patients. *Fusobacterium* is a typical colorectal cancer-related pathogen [27,28]. *Fusobacterium* was also found to be more abundant in diabetes patients than in non-diabetic subjects [29,30]; as such, the role of *Fusobacterium* concerning the pathogenesis of RT2D needs further study. *Veillonella* is a gram-negative anaerobic cocci that normally resides in the gastrointestinal tract, oral cavity and vagina [31,32]. An increased abundance of *Veillonella* was detected in the saliva of the oral microbiome in T2D and gestational diabetes mellitus, as well as a pathogen in diabetic patients with osteomyelitis [33,34,35]. The species of *Veillonella denticariosi* (*V. denticariosi*) was first isolated from human carious dentine, and the function of *V. denticariosi* remained uncertain [36]. Therefore, this study showed that *V. denticariosi* has a potential role in diabetes.

We also found that the abundance of *A*. *muciniphila* (T2D: 1.25%; RT2D: 0.09%, *p* = 0.04) was significantly reduced while *Bacteroides vulgatus* (T2D: 8.68%; RT2D: 14.34%, *p* = 0.03) was significantly increased in RT2D patients. *Bacteroides vulgatus* was identified as the major species associated with the biosynthesis of branched-chain amino acids (BCAAs) and increased insulin resistance [37]. Additionally, in this study, all subjects received the first-line standard therapy of Metformin except for patients with decreased renal function. Metformin is a biguanide that is in wide use for the treatment of T2D as a first-line OGLD. Several mechanisms have been proposed to explain the actions of Metformin, including decreased gluconeogenesis, the absorption of glucose from the gastrointestinal tract and increased insulin sensitivity and peripheral glucose uptake [38]. Interestingly, Metformin also alters the gut microbiota by way of its association with an increased abundance of mucin-degrading *A. muciniphila* as a means to promote glucose homeostasis [39,40]. However, in our studies, both groups of T2D and RT2D received Metformin therapy, and the abundance of *A*. *muciniphila* was still significantly reduced in refractory diabetes, indicating that the alteration of *A*. *muciniphila* was not affected by Metformin in refractory diabetes.

There is currently growing interest in the role and function of *A. muciniphila*, as it has been recognized as a probiotic that may be specifically effective for use in diabetes [41]. In a mouse model, the administration of either pasteurized *A. muciniphila* or its outer-membrane protein Amuc_1100* activated Toll-like receptor 2 (TLR2) and increased the expression of the tight-junction proteins, which in turn reversed high-fat diet (HFD)-induced obesity and reduced insulin resistance [42]. *A. muciniphila* may also mediate the negative effects of interferon-gamma (IFNγ) on glucose tolerance in IFNγ-deficient mice and reduce hepatic glycogen in streptozotocin-induced diabetic rats [43,44]. In humans, a decreased abundance of *A. muciniphila* was also observed in newly-diagnosed patients with prediabetes as well as those with T2D [41,45,46,47]. Most importantly, we found that the relative proportion of *A*. *muciniphila* was significantly negatively correlated with HbA1c. Good glycemic control is critical for T2D patients, as reductions in HbA1c are directly associated with a reduced risk of microvascular/macrovascular complications [48,49]. Our findings were identical to those reported for human subjects in the Advento study [43]. As such, reductions in the abundance of the glucose homeostasis-related species, *A. muciniphila*, together with an increased abundance of the insulin resistance-related species, *B. vulgatus*, may have an overall impact on the therapeutic effect and the mechanisms underlying the development of RT2D.

We further found that these microbial markers were involved in several glucose-related functional pathways. Compared with T2D, the reduction of the biosynthesis of unsaturated fatty acids has an adverse impact on fasting blood glucose and insulin levels [50]. It has been reported in various studies that arachidonic acid (AA) has a positive effect on stimulating glucose uptake and that reductions in AA metabolism may have a direct impact on insulin-stimulated glucose uptake [51,52,53]. By contrast, the phosphotransferase system (PTS) utilizes phosphoenolpyruvate (PEP) as a phosphoryl donor for sugar phosphorylation and is involved in the transport of sugars into bacteria. The increase of the phosphotransferase system has been reported in prediabetes and gestational diabetes mellitus [54,55] and further enriched in refractory diabetes in our studies. These alterations of functional pathways may have contributed to the development of refractory diabetes.

The early evaluation of and aggressive interventions for glycemic control are critical in refractory diabetes. Clinical observations that are typically used to predict RT2D include early age of onset, longer duration of diabetes and the presence of microvascular/macrovascular complications, as well as insulin use and the overall complexity of the therapeutic regimen [10]. Our studies reveal potential markers within the gut microbiota that can distinguish RT2D from T2D (AUC = 0.719, *p* < 0.01), and the examination of the gut microbiota could evaluate the ability of glycemic control and the therapeutic results of diabetes patients. Additionally, microbiome intervention/modulation may offer another therapeutic approach for refractory diabetes patients. Probiotics including certain genera of *Lactobacillus* and *Bifidobacterium* are currently commonly used as human nutrition supplements and meet the goal of a positive effect on human health. In T2D patients, probiotic supplements have been widely studied, and several trials have demonstrated that probiotic intervention could improve fasting blood glucose, HbA1c and insulin sensitivity [56,57]. As a promising probiotic, *A. muciniphila* is also currently under investigation for use in various diseases, including diabetes [41,58,59].

The present study has certain limitations, as the subject numbers in this study were quite small, and larger studies involving more participants are required to solidify the value of the gut microbiota in distinguishing refractory diabetes patients from T2D. However, our findings reveal a new possibility of the gut microbiota in refractory diabetes and provide clinical implications that evaluate the ability of glycemic control and provide therapeutic strategies taking the gut microbiota into account in the future.

## 5. Conclusions

In conclusion, this work presents a novel study that elucidates the gut microbiota in refractory diabetes patients. The alteration of the gut microbiota pattern could distinguish refractory diabetes from diabetes with good glycemic control. Additionally, the abundance of *Akkermansia muciniphila* was significantly negatively correlated to HbA1c. These results indicated that the examination of the gut microbiota could be used to evaluate glycemic control in diabetes patients. Microbiome intervention and modulation could be used as a therapeutic strategy for improving glycemic control of refractory diabetes in the future.

## Figures and Tables

**Table 1:** Characteristics of the study subjects with type 2 diabetes (T2D) and refractory diabetes (RT2D).

	T2D, N = 52	RT2D, N = 27
Age (years)	66.38 ± 1.314	64.37 ± 2.194
Gender (M/F)	29/23	16/11
HbA1c (%)	7.03 ± 0.079	8.99 ± 0.214
Glucose AC (mg/dL)	135.1 ± 3.283	158.9 ± 13.67
Triglyceride (mg/dL)	150.7 ± 11.23	135.7 ± 14.07
Total cholesterol (mg/dL)	181.6 ± 4.861	178.8 ± 5.251
HDL (mg/dL)	47.38 ± 2.373	45.99 ± 2.644
LDL (mg/dL)	113.4 ± 4.161	109.3 ± 5.642
Creatinine (mg/dL)	1.02 ± 0.041	1.08 ± 0.06
